# Post-thoracotomy paraplegia after oxidized cellulose spinal compression

**DOI:** 10.31744/einstein_journal/2023RC0078

**Published:** 2023-06-21

**Authors:** Da Jun Than, Vinodh Vayara Perumall, Syamim Johan, Xin Leh Lee, Khasnizal Abd Karim, Firdaus Hayati

**Affiliations:** 1 Queen Elizabeth Hospital Ministry of Health Malaysia Kota Kinabalu Sabah Malaysia Queen Elizabeth Hospital, Ministry of Health Malaysia, Kota Kinabalu, Sabah, Malaysia.; 2 Faculty of Medicine and Health Sciences Universiti Malaysia Sabah Kota Kinabalu Sabah Malaysia Faculty of Medicine and Health Sciences, Universiti Malaysia Sabah, Kota Kinabalu, Sabah, Malaysia.; 3 Tawau Hospital, Ministry of Health Malaysia Tawau Sabah Malaysia Tawau Hospital, Ministry of Health Malaysia, Tawau, Sabah, Malaysia.

**Keywords:** Hemostasis, Iatrogenic disease, Paraplegia, Thoracotomy, Cellulose, oxidized, Spinal cord compression

## Abstract

Post-thoracotomy paraplegia after non-aortic surgery is an extremely uncommon complication. A 56-year-old woman presented with a 1-year history of progressive shortness of breath. Computed tomography revealed a locally advanced posterior mediastinal mass involving the ribs and the left neural foramina. Tumor excision with a left pneumonectomy was performed. Post-resection, bleeding was noted in the vicinity of the T4-T5 vertebral body, and the bleeding point was packed with oxidized cellulose gauze (Surgicel^®^). Postoperatively, the patient complained of bilateral leg numbness extending up to the T5 level, with bilateral paraplegia. An urgent laminectomy was performed, and we noted that the spinal cord was compressed by two masses of Surgicel^®^ with blood clots measuring 1.5 × 1.5cm at T4 and T5 levels. The paraplegia did not improve despite the removal of the mass, sufficient decompression, and aggressive postoperative physiotherapy. Surgeons operating in fields close to the intervertebral foramen should be aware of the possible threat to the adjacent spinal canal as helpful hemostatic agents can become a preventable threat.

## INTRODUCTION

Post-thoracotomy paraplegia in non-aortic surgery is an extremely uncommon complication, with an estimated incidence of 0.08%.^(1)^ Post-thoracotomy paraplegia caused by hematoma and oxidized cellulose compression is a rare but devastating complication, with 15 cases reported between 1984 and 2017.^(2,3)^ In this report, we present a case of paraplegia caused by spinal compression by Surgicel^®^ after resection of thoracic chondrosarcoma.

## CASE REPORT

A 56-year-old female with a 1-year history of left-sided chest pain and worsening shortness of breath was admitted to our hospital for a workup. Contrast-enhanced computed tomography of the thorax showed a posterior mediastinal mass measuring 17 × 25 × 9cm with no clear plan between the posterior 4^th^ and 5^th^ rib as well as the T4 and T5 left neural foramina. Surgery was performed under general anesthesia using a double-lumen endobronchial tube. After single-lung ventilation was established, the patient was placed in the right lateral decubitus position. Thoracotomy via a posterolateral skin incision was performed through the 5^th^ intercostal space. Intraoperatively, a tumor invading the left upper and lower lobes of the lung was noted; hence, a decision was made for tumor excision with left pneumonectomy, and the tumor part that invaded the neural foramina was easily removed with minimal dissection. However, after tumor removal, bleeding from the neural foramina of the T4 and T5 vertebral bodies was observed, which was difficult to control despite the use of electrocautery. An oxidized cellulose gauze (Surgicel^®^) was packed into the T4 and T5 neural foramina to achieve hemostasis.

**Figure 1:**
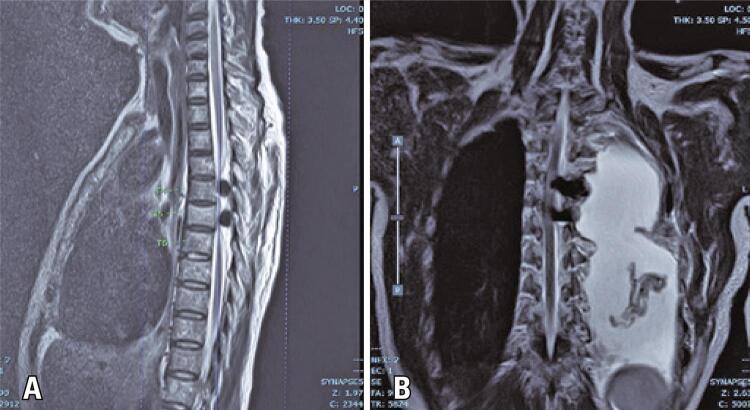
Sagittal (A) and coronal (B) T2 magnetic resonance imaging showing T4 and T5 hypointense mass

**Figure 2:**
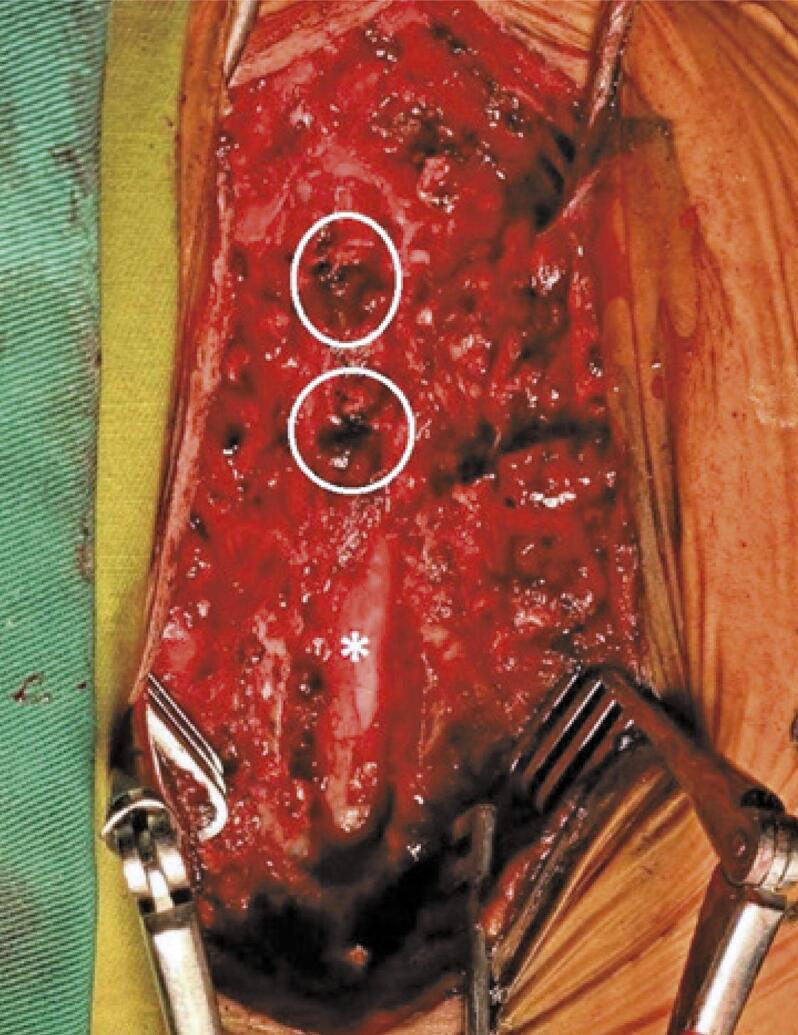
Surgicel® was placed at the T4 and T5 levels (circles). The spinal cord (asterisk) is visible

**Figure 3:**
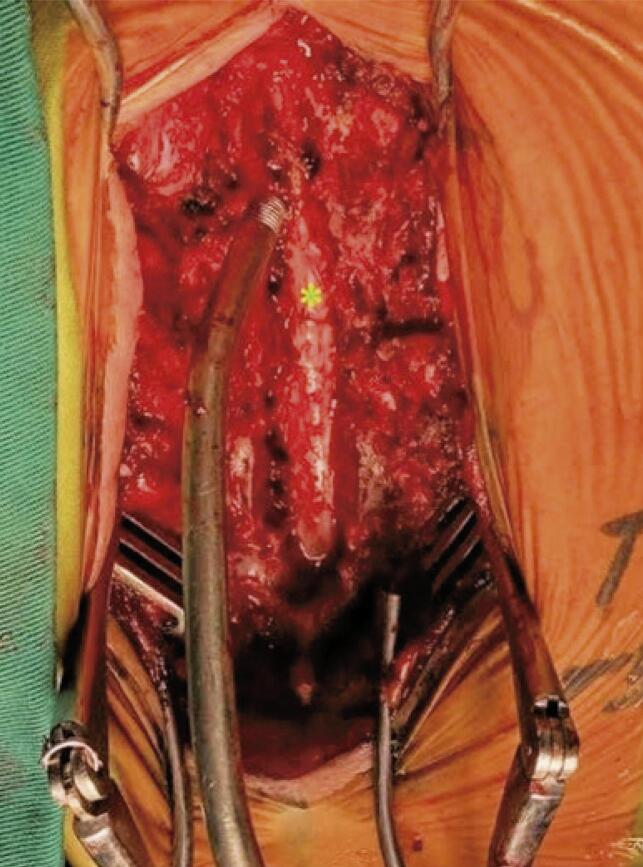
The spinal cord (asterisk) after a decompression

The patient was admitted to the intensive care unit. Upon awakening from anesthesia, she complained of bilateral lower limb numbness extending up to the T5 level, with bilateral paraplegia. At a 12-hours postoperative period, magnetic resonance imaging (Figure 1) was performed. Magnetic resonance imaging revealed an extradural compressive localized mass at T4 and T5 that extended via the intervertebral foramen. An urgent laminectomy was performed, and we found that the spinal cord was compressed by two masses of Surgicel^®^ with blood clots measuring 1.5 × 1.5cm at the T4 and T5 level (Figure 2). The mass was removed by sufficient decompression (Figure 3). Unfortunately, despite aggressive physiotherapy, the paraplegia did not improve, except for minimal sensory recovery.

## DISCUSSION

These reports share many features with our case, namely, posterolateral thoracotomy with difficulty in achieving hemostasis near the paravertebral area despite using diathermy. Surgicel^®^ was used, left in situ, and was later found within the spinal canal during exploration. Oxidized cellulose is commonly used as a hemostatic agent. Once soaked in blood, it expands into a gelatinous mass, which aids in the formation of a clot, possibly exerting a compressive effect on the surrounding structures. This case report discusses the potential risk of iatrogenic spinal cord injury when oxidized cellulose is used as a hemostatic agent near the neural foramina region.

The spinal canal is only a few millimeters from the intrathoracic opening of the intervertebral foramen.^(4)^ Although there is no connection between the pleural cavity and epidural space under normal circumstances, disruption during surgery may create such connections.^(5)^ Some authors have postulated that in the presence of a connection, the pressure difference between these two cavities drives oxidized cellulose to migrate into the spinal canal through the intervertebral foramen, causing spinal cord compression.^(4-6)^ These studies also stipulated that the most difficult bleeding occurred near the end of the posterior rib, and in all cases, bleeding was only controlled with oxidized cellulose after attempts at pressure and diathermy.^(4-7)^

Paraplegia after thoracotomy for non-aortic surgery requires urgent investigation and intervention to salvage spinal cord function and prevent subsequent morbidity. Magnetic resonance imaging revealed two well-defined hypointense masses on T1- and T2-weighted images. These characteristics are similar to the previously reported case as it also emphasizes the difference of contrast appearance between hematomas which appears as hyperintense and spread out in comparison to Surgicel^(6)^ Thus, in the setting of neurological deficits after thoracotomy, especially in instances where compressive hemostatic agents are left *in situ*, the surgeon must always have a high index of suspicion and act immediately to obtain both neurological consultation and imaging. If decompression is performed within 8 hours after the symptoms are described, the incidence of paraplegia is approximately 20%, whereas after 24 hours, the risk of paraplegia can increase to 60%.^8^

## CONCLUSION

Surgeons operating in the thoracic cavity near the intervertebral foramen should be aware of the possible threat to the adjacent spinal canal because a meticulous surgical technique with good visualization of the operative field is imperative to reduce the risk of excessive bleeding. Resection of the ribs is necessary to salvage the operative field. In cases where Surgicel^®^ are used as hemostatic agents, attempts should be made to remove them all to avoid any serious and preventable complications.
